# Moderate Grazing Promotes Arthropod Species Diversity in an Alpine Meadow

**DOI:** 10.3390/biology12060778

**Published:** 2023-05-27

**Authors:** Huanhuan Guan, Shangyun Zhang, Yifei Huangpu, Han Yan, Karl J. Niklas, Tserang Donko Mipam, Shucun Sun

**Affiliations:** 1 Department of Ecology, School of Life Sciences, Nanjing University, 163 Xianlin Avenue, Nanjing 210023, China; dg1830050@smail.nju.edu.cn (H.G.); mg20300075@smail.nju.edu.cn (S.Z.); mg20300040@smail.nju.edu.cn (Y.H.); mg21300075@smail.nju.edu.cn (H.Y.); 2 School of Integrative Plant Science, Cornell University, Ithaca, NY 14853, USA; kjn2@cornell.edu; 3 Sichuan Zoige Alpine Wetland Ecosystem National Observation and Research Station, Institute of Qinghai-Tibetan Plateau, Southwest Minzu University, Chengdu 610041, China

**Keywords:** grazing intensity, arthropod, species diversity, intermediate disturbance hypothesis, Alpine meadow

## Abstract

**Simple Summary:**

Plant species diversity has been substantially studied in relation to livestock grazing intensity. However, the effect of grazing on arthropod species diversity has seldom been addressed. Here, we report the results of a two-year plant and arthropod survey from 2020 to 2021 for four levels of grazing intensity, including nongrazing (as a control), light grazing, moderate grazing, and heavy grazing, of the long-term grazing experiment starting in 2016. We found that the species richness and diversity of the major arthropod species groups (including herbivores, parasitoids, and detritivores, including both saprophages and coprophages) peaked (albeit statistically non-significantly in specific years) in the moderate grazing treatment, whereas predator species richness and diversity did not differ significantly among the different treatments. The data indicate that the relationship between grazing intensity and arthropod diversity is consistent with the intermediate disturbance hypothesis, which postulates that moderate grazing should be applied in pastures to maximize multi-functional ecosystem services.

**Abstract:**

Livestock grazing is an important tool used in grassland land management practices. Studies have substantially addressed the effect of grazing on plant species diversity, revealing that moderate grazing increases plant species diversity. However, few studies have dealt with the relationship between grazing and arthropod species diversity, which remains unclear. Here, we hypothesize that moderate grazing promotes arthropod species diversity because arthropods are directly or indirectly dependent on plant diversity. In this study, we conducted a two-year plant and arthropod survey from 2020 to 2021 at four levels of grazing intensity, i.e., nongrazing (as a control), light grazing, moderate grazing, and heavy grazing, of the long-term grazing experiment starting in 2016. The data show that plant species diversity peaked in the moderate grazing treatment, and herbivore species diversity was positively correlated with plant species diversity (and hence peaked in the moderate grazing treatment). Moderate grazing promoted parasitoid species diversity, which was positively correlated with herbivore species diversity. However, predator species diversity did not significantly differ among the four treatments. In addition, saprophage species diversity decreased, whereas coprophages increased with increasing grazing levels, such that species richness (but not species diversity of detritivores statistically) was highest in the moderate grazing treatment. Consequently, the species diversity of arthropods as a whole peaked at the moderate grazing level, a phenomenology that is consistent with the intermediate disturbance hypothesis. Considering that moderate grazing has been found to increase plant species diversity, facilitate soil carbon accumulation, and prevent soil erosion, we suggest that moderate grazing would maximize multi-functional ecosystem services.

## 1. Introduction

Grasslands cover ~40% of the Earth’s land surface, and most are used as pastures [1,2] that not only produce commercially important dairy products but also provide multi-functional ecosystem services, including maintaining biodiversity, regulating climate, preventing soil erosion, and protecting water resources [3,4]. Grazing intensity is often controlled to optimize pasture multi-functional ecosystem services [5]. For example, moderate grazing can increase fodder yields, soil organic matter accumulation, and nutrient cycling in many pastures [6,7,8]. In particular, plant species diversity is reported to peak under moderate grazing but not heavy or light grazing [9], consistent with the intermediate disturbance hypothesis [10]. However, the effect of grazing intensity on invertebrate (e.g., arthropod) species diversity has been much less studied. Indeed, how grazing affects animal diversity, in general, has been much less explored [11].

Arthropods are the most abundant and diverse group of invertebrates in pastures and play critical and diverse ecosystem roles and functions [12]. For example, herbivorous insects can significantly reduce plant resources for livestock [13,14,15], whereas parasitoid and predator species can favor both plant species abundance and diversity by limiting the number of herbivore species [16,17]. Moreover, detritivores such as springtails and dung beetles can facilitate the decomposition of plant litter and livestock dung [18,19,20]. As all of the ecosystem functions of arthropods are associated with livestock, the grazing intensity of livestock may in turn affect the abundance and species diversity of arthropods [21,22,23,24].

In this context, it is widely observed that herbivorous species diversity is positively correlated with plant species diversity, perhaps because more types of resources tend to support more diverse consumer species [25]. Livestock grazing affects the species diversity of herbivorous insects, presumably by changing plant species diversity [26,27]. However, the relationship between grazing intensity and plant diversity is not invariably linear. For example, several studies report that high and low grazing as well as non-grazing can reduce plant species diversity, whereas moderate grazing can increase plant species diversity [28]. This intermediate disturbance phenomenon, which favors plant species diversity, has been reported in many types of grasslands [29].

Thus, we hypothesize that moderate grazing would increase herbivorous arthropod species diversity. Similarly, it has been hypothesized that secondary consumers, which primarily prey on herbivores, should follow a similar trend to herbivorous insect species [30], presumably because more prey species can provide more niches for more parasitoid and/or predator species [31]. For example, higher numbers of parasitoid species are often associated with larger numbers of herbivorous insect species, principally because most parasitoid species are host-species-specific [32]. It is also reported that more prey species can support more predator species, likely because many predators are prey species-specific [33]. Therefore, we hypothesize that moderate grazing would promote the species diversity of secondary consumers, including parasitoids and predators.

In addition, grazing may also significantly affect the species diversity of detritivores, including saprophages primarily based on its indirect effects on plant litter (e.g., springtails) and coprophages primarily utilizing livestock dung (e.g., dung beetles) [34,35]. According to the resource abundance hypothesis, consumer species diversity is expected to positively correlate with resource abundance, such that the species diversity of saprophages may decrease with increasing grazing intensity as heavy grazing devours most green plant parts, leaving less plant litter [36]. Similarly, the species diversity of coprophages may increase with increased grazing intensity as higher livestock can produce more dung resources [37]. It is, therefore, possible that both saprophages and coprophages occur with an intermediate level of species richness and diversity at moderate grazing intensity, likely leading to a higher species diversity of detritivores [38]. Thus, we hypothesize that moderate grazing would promote the species diversity of detritivores, including saprophages and coprophages.

If the functional groups of arthropods will increase in diversity under moderate grazing intensity, it is logical to conclude that moderate grazing will promote arthropod species diversity overall in pastures. However, previous studies have either investigated only two stocking rates [39] or investigated specific functional groups (e.g., dung beetles) or specific arthropod taxa, e.g., Orthoptera, Lepidoptera, and Diptera [40,41,42], such that few studies have tested whether moderate grazing promotes the overall species diversity of arthropods.

To test the aforementioned hypotheses, we conducted a grazing experiment involving four grazing intensities (i.e., nongrazing control, light grazing, moderate grazing, and heavy grazing) in an alpine meadow in the eastern Tibetan Plateau. We recorded the relative abundance for each arthropod and plant species, and we determined whether: (1) moderate grazing increases plant species diversity; (2) moderate grazing increases species diversity for each arthropod functional group (including herbivores, parasitoids, predators, and detritivores).

## 2. Methods and Materials

### 2.1. Study Site

This study was conducted at Hongyuan Alpine Meadow Station (32°48′ N, 102°33′ E; 3500 m above sea level) of Nanjing University, located in the eastern part of the Qinghai-Tibet Plateau. The average annual temperature is 1.7 °C, with the highest and lowest monthly averages occurring in July and January (i.e., 11.1 °C and −9.3 °C, respectively), according to the records between 1961 and 2019 of the Hongyuan County Meteorological Station (about 5.0 km from the study site). The average annual precipitation is ~756 mm, with large fluctuations between years, with 80% occurring from May to September [43].

The alpine meadow has over 90% vegetation cover, with an average plant height of ~30 cm. The vegetation is dominated by forbs such as *Saussurea nigrescens* Maxim., *Anaphalis flavescens* Hand.-Mazz., *Polygonum viviparum* Linn., and *Potentilla anserine* (L.) Rydb.; sedges such as *Kobresia myosuroides* (Villars) Fiori, and *Carex* spp.; and grasses such as *Deschampsia caespitosa* (L.) P. Beauv., *Koeleria litvinowii* Dom., *Festuca ovina* L., and *Elymus nutans* Griseb. [44].

### 2.2. Experimental Design

In April 2014, a representative and relatively uniform grassland (ca. 20 ha in area) was selected for the alpine meadow study site. Nine grazing plots (each with an area of 1 ha of 100 m × 100 m) and three nongrazing (UG) control plots (each with an area of 0.33 ha of 55 m × 60 m) were constructed and divided using net fences. Grazing was prohibited on each plot during the subsequent months of 2014. The grazing experiment was initiated in May 2015. Three grazing plots were assigned to each of the light (LG), moderate (MG), and heavy grazing (HG) treatments. The stocking rates were one, two, and three yaks (*Bos grunniens* Linnaeus) per hectare for LG, MG, and HG, respectively. The body weight of each yak was approximately 200 kg. Grazing intensity levels were achieved in the following ways: During the growing season (from late May to late September each year), one, two, and three yaks were moved to graze in each of the LG, MG, and HG replicate treatments, respectively. Grazing was restricted from 07:00 to 19:00 each day, after which the yaks were quickly returned to a barn. For a complete and detailed experimental design, see Mipam et al., 2019 [44,45].

### 2.3. Plant Sampling

Plant biomass was measured in mid-August, whereupon six 50 × 50 cm quadrats were randomly selected from each test plot, and the abundance, frequency, cover, and height of each plant species were recorded in each quadrat. The aboveground plant parts of four functional plant groups (i.e., grasses, sedges, legumes, and forbs) were subsequently harvested, dried at 65 °C for 72 h, and weighed.

### 2.4. Arthropod Sampling and Identification

Arthropods were collected once each month from June to September in 2020 and 2021. To obtain samples as comprehensively as possible, three sampling methods were used (i.e., sweeping nets, yellow disks, and pitfall traps). Sweeping nets were used to catch arthropods in the air and on plants, including butterflies, dragonflies, bees, mosquitoes, flies, leafhoppers, leaf beetles, and web-building spiders. Sweeping was conducted on sunny days between 11:00 and 15:00. In each grazing plot, sweeping was conducted three times using sweeping nets with a pole length of 1.5 m, each time advancing 33 nets at a constant speed; in each nongrazing plot, each advance had 11 nets. All the sweepings were accomplished in the middle of each plot, at a distance of at least 20 m from the perimeter [46].

Yellow discs were used to collect insects with a yellow preference, such as butterflies, flies, bees, and planthoppers. Yellow disks were placed before the fog cleared at 6 a.m. on sunny days and collected at 6 p.m. before sunset. Several drops of detergent were added to the water in each disk to reduce surface tension. In each grazing plot, 3 yellow disks were randomly placed on the ground at a distance of at least 20 m from the perimeter of each plot; each yellow disk was 10 m apart; in the nongrazing plot, only 1 yellow disc was placed [47].

Pitfall traps were used to collect arthropods inhabiting the ground surface, such as beetles, coprophagus, staphylinids, and run-spiders. Each trap consisted of 415-mL plastic containers filled with 200 mL water. The traps were collected after three consecutive days. In each plot, three pitfall traps were randomly placed at least 10 m apart below the ground surface at a distance of at least 20 m from the perimeter of each plot [48].

All samples were sorted in the laboratory to produce one set of dry specimens. The rest of the collection was bottled with 95% alcohol immersion. All arthropods were identified to the species level by entomologists when morphological features were unambiguous, whereas species with ambiguous or debatable features were identified using DNA barcoding.

All the collected arthropods were divided into four feeding habit guilds, i.e., herbivores, detritivores, parasitoids, and predators [49,50]. The detritivore guild included coprophages feeding on dung and saprophages that primarily consume plant litter and soil humus.

### 2.5. Statistical Analysis

The species obtained by yellow disk collection were removed if they overlapped with those obtained by the sweeping net. The remaining species were pooled with those obtained by the sweeping and trapping methods, following the protocols of previous studies using different arthropod sampling methods [51,52]. As the phenologies of different arthropod species can differ, we calculated the average value of the four months’ collections for each replicate plot in both 2020 and 2021 in order to obtain sufficient statistics. Additionally, as there was no buffer zone between different plots, three insect species with high flight abilities (including the butterfly *Colias fieldii*, the adult moth *Macdunnoughia crassisigna,* and the dragonfly *Brachythemis contaminata*) were excluded from statistical analyses.

All the data were tested to determine their frequency distributions, and a logarithmic transformation was performed on the plant and arthropod richness data that did not show a normal distribution. To test whether yak grazing intensity affects plant and arthropod diversity (species richness and Shannon–Wiener index), one-way analysis of variance (ANOVA) was used, followed by Fisher’s LSD test for *post hoc* comparison, which was used because of the small number of replicates (*n* = 3) for each treatment. A linear model “lm” was used to determine the relationships between plant and arthropod diversity. All data analysis was conducted in R 4.0.3.

## 3. Results

### 3.1. Plant

**Figure 1:**
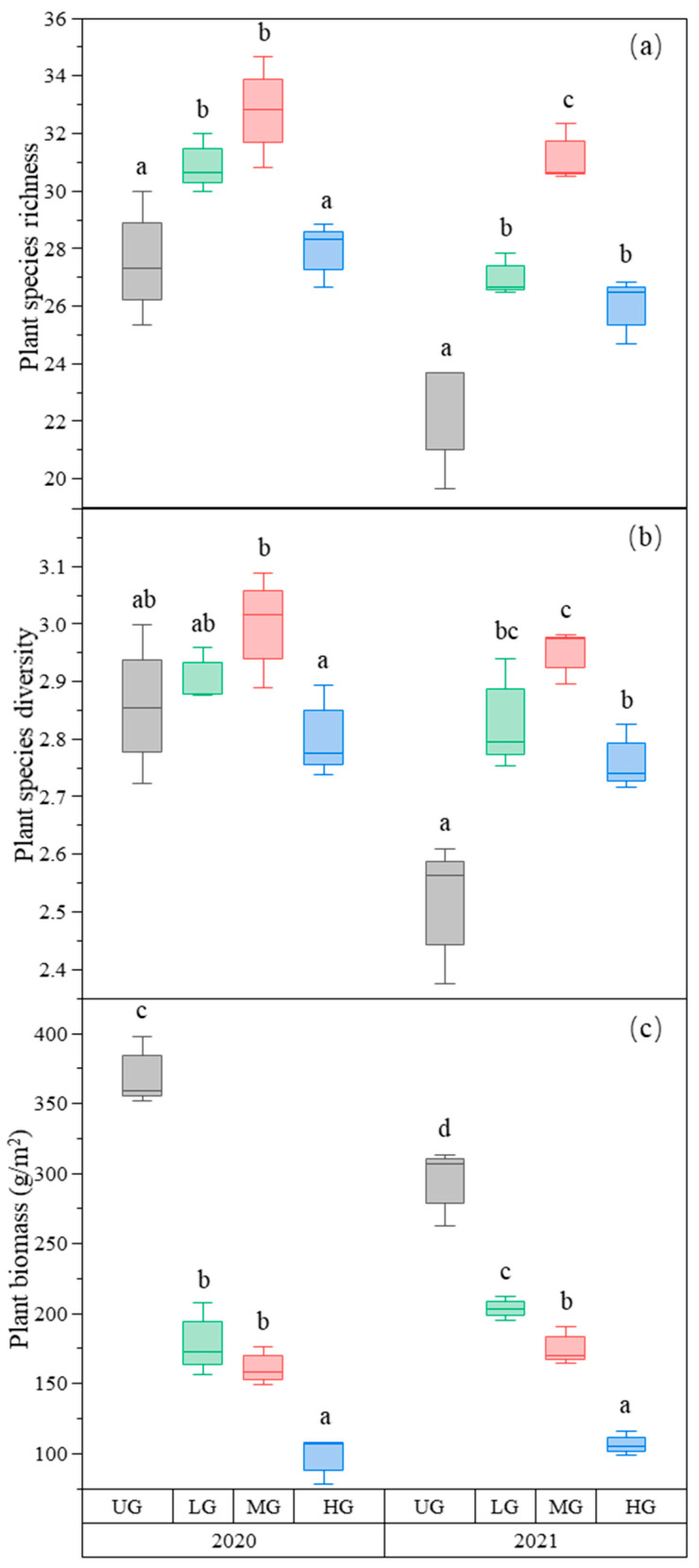
Box plot of plant species richness (**a**); plant species diversity (**b**); and plant biomass (g/m^2^) (**c**) for the nongrazing (UG), light (LG), moderate (MG), and heavy (HG) grazing treatments. The error bars represent the standard error; the letters represent the significant differences (*p* < 0.05).

In our study site, 6 grass species, 4 sedge species, 3 legume species, and 52 forb species were found. Plant species richness was the highest in the MG treatment and higher in the LG treatment compared to the UG and HG treatments (Figure 1a, Table 1). Plant species diversity (Shannon–Wiener index) was also the highest in MG, whereas LG and HG were higher than UG in 2021. However, there was no significant difference among the three grazing intensities in 2020 (Figure 1b, Table 1). As expected, the aboveground plant biomass decreased with increasing grazing intensity (Figure 1c, Table 1). However, although the relative biomass of the grass species group decreased, the relative biomass of the forb species group increased with increasing grazing intensity (Table 2).

### 3.2. Herbivores

A total of 48,853 individuals from 282 species, 9 orders, 89 families, and 2 classes of arthropods were identified over 2 years.

**Figure 2:**
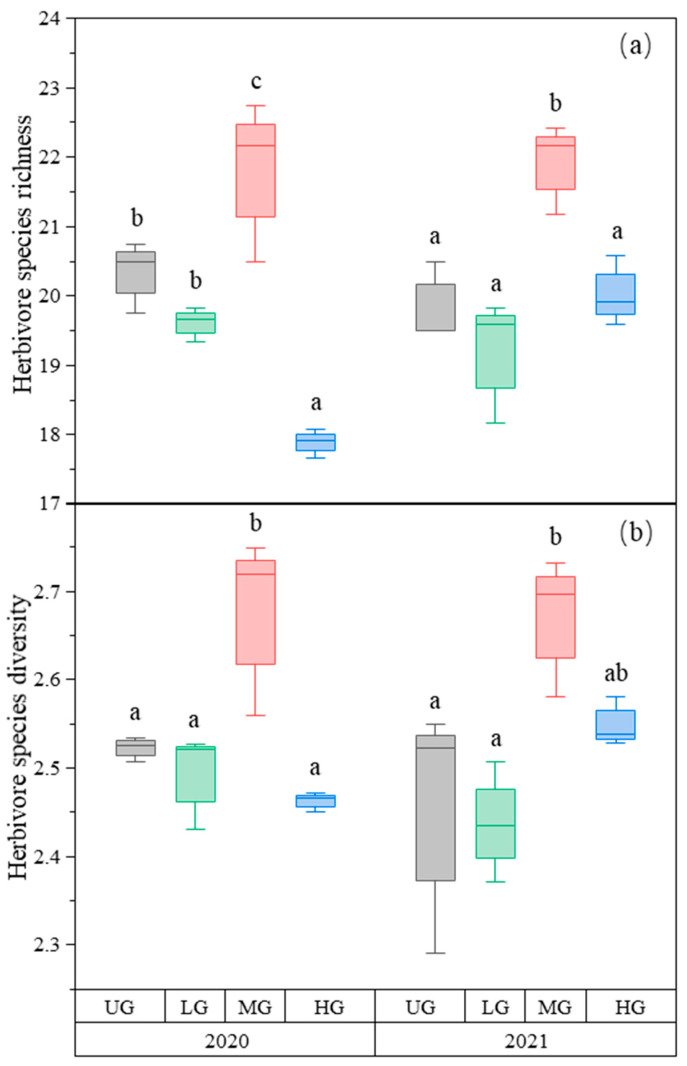
Box plot of herbivore species richness (**a**) and species diversity (**b**) of the nongrazing (UG), light (LG), moderate (MG), and heavy (HG) grazing treatments in 2020 and 2021. The error bars in the figure represent the standard error, and the letters represent the significant differences (*p* < 0.05).

The species richness of herbivores was the highest in the MG treatment compared to the other treatments. The species richness in HG was lower than in UG and LG in 2020. There was no difference among the three treatments in 2021 (Figure 2a, Table 1). Similarly, herbivore species diversity was highest in MG, but there was no difference among UG, LG, and HG (Figure 2b, Table 1).

### 3.3. Parasitoids and Predators

**Figure 3:**
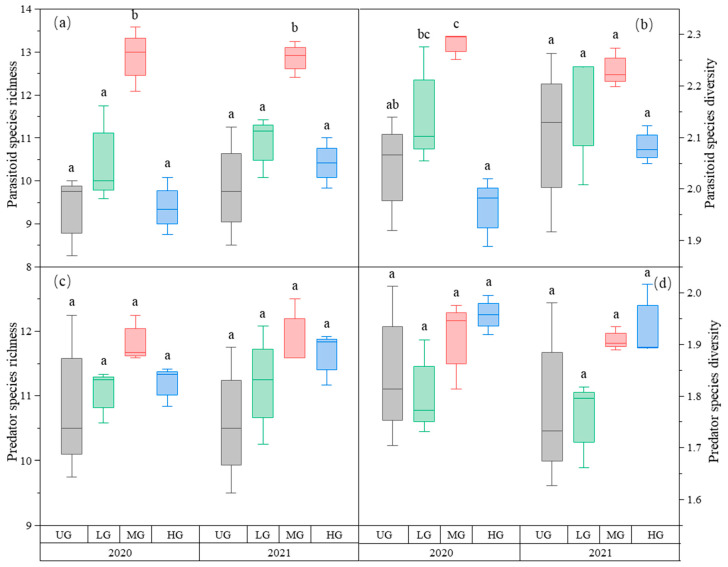
Box plot of parasitoid species richness (**a**); parasitoid species diversity (**b**); predator species richness (**c**); and predator species diversity (**d**) at nongrazing (UG), light (LG), moderate (MG), and heavy (HG) grazing treatments in 2020 and 2021. The error bars in the figure represent the standard error; the letters represent the significant differences (*p* < 0.05).

Parasitoid richness was the highest in the MG treatment. There was no difference in parasitoid richness among the three UG, LG, and HG treatments (Figure 3a, Table 1). The parasitoid species diversity in MG was highest in 2020 but not in 2021. Grazing had no significant effect on parasitoid species diversity in 2021 (Figure 3b, Table 1). The species richness and diversity of predators did not differ significantly among the three grazing intensities (Figure 3c,d, Table 1).

### 3.4. Detritivores

**Figure 4:**
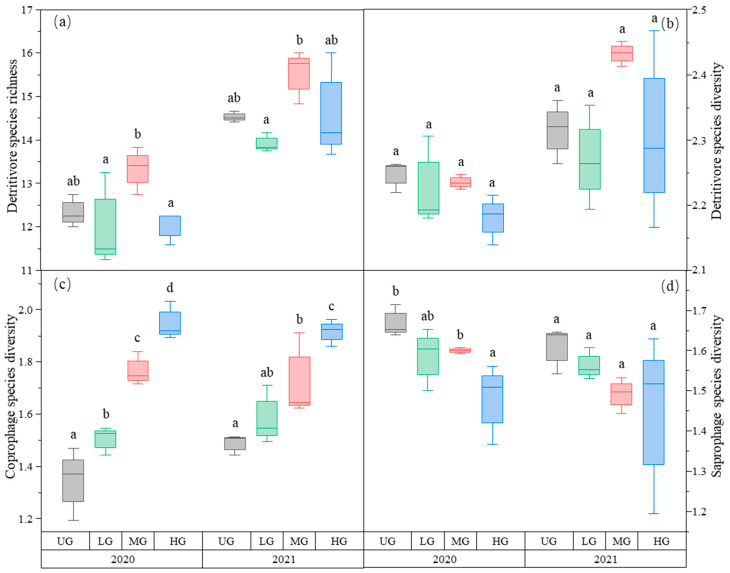
Box plot of detritivore species richness (**a**); detritivore species diversity (**b**); coprophage species diversity (**c**); and saprophage species diversity (**d**) of the nongrazing (UG), light (LG), moderate (MG), and heavy (HG) grazing treatments in 2020 and 2021. The error bars in the figure represent the standard error, and the letters represent the significant differences (*p* < 0.05).

The species richness of detritivores was higher in the MG treatment than in the LG treatment. There was no difference among the three UG, LG, and HG treatments (Figure 4a, Table 1). In contrast, detritivores’ species diversity did not differ among the three different grazing intensities (Figure 4b, Table 1).

Among the detritivores, the species diversity of coprophages increased with increasing grazing intensities (Figure 4c, Table 1), whereas that of saprophages decreased with increasing grazing intensity in 2020 but not in 2021 (Figure 4d, Table 1).

### 3.5. Overall Effects on Arthropods

**Figure 5:**
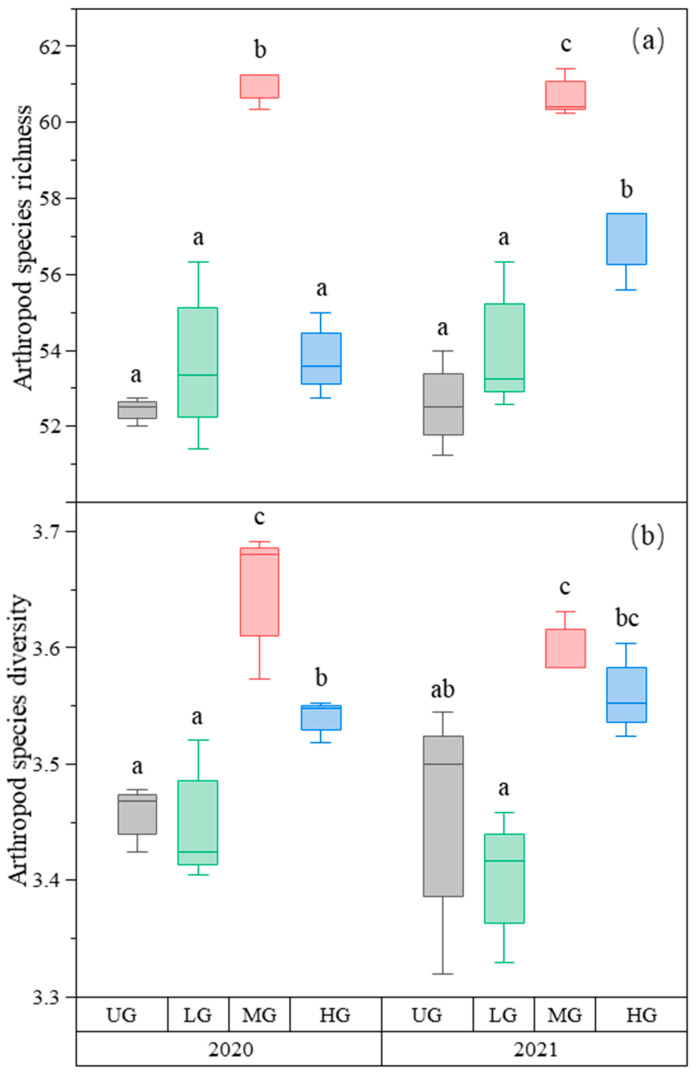
Box plot of arthropod species richness (**a**) and species diversity (**b**) for nongrazing (UG), light (LG), moderate (MG), and heavy (HG) grazing treatments in 2020 and 2021. The error bars in the figure represent the standard error, and the letters represent the significant differences (*p* < 0.05).

Arthropod species richness was highest in the MG treatment compared to the other treatments, and that in the HG treatment was higher than in the UG and LG treatments in 2021, but there was no difference between UG, LG, and HG in 2020 (Figure 5a, Table 1). Arthropod species diversity was also the highest in the MG treatment compared to the other grazing intensities, and HG was higher than UG and LG (Figure 5b, Table 1).

### 3.6. Relationships among Plant, Herbivore, and Parasitoid Species Diversity

**Figure 6:**
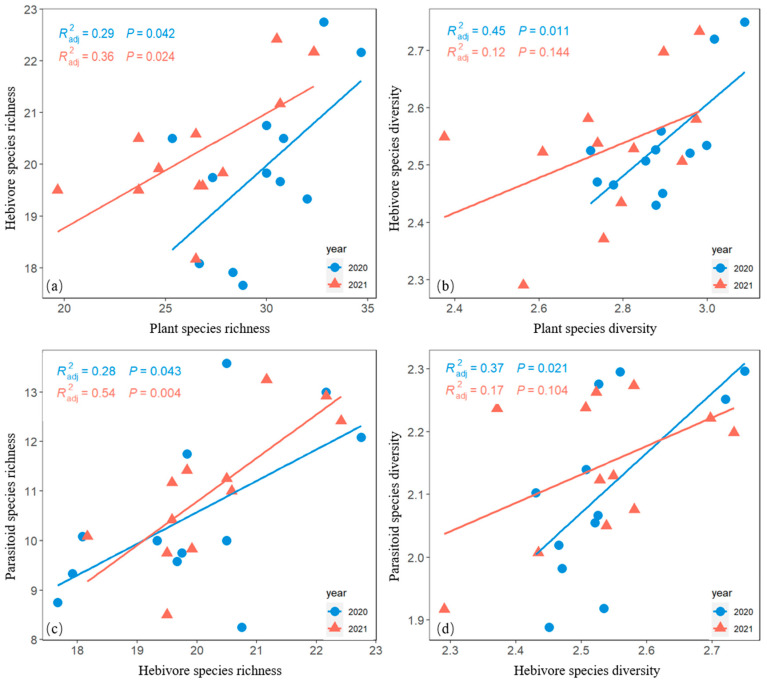
Bivariate-plot relationships of herbivore species richness versus plant species richness (**a**), herbivore species diversity versus plant species diversity (**b**), parasitoid species richness versus herbivore species richness (**c**), and parasitoid species diversity versus herbivore species diversity (**d**) in both 2020 and 2021.

Herbivore richness was positively correlated with plant species richness (Figure 6a). Herbivore species diversity was positively correlated with plant species diversity in 2020 but not with plant species diversity in 2021 (Figure 6b).

Parasitoid species richness was positively correlated with herbivore species richness (Figure 6c). Parasitoid species diversity was positively correlated with herbivore species diversity in 2020 but not with herbivore species diversity in 2021 (Figure 6d).

## 4. Discussion

The results presented here indicate that grazing intensity affects both plant and arthropod species diversity and that moderate grazing promotes both the species richness and diversity of both plants and arthropods. Specifically, the species richness and diversity of herbivores, parasitoids, and detritivores are seen to peak at the moderate grazing treatment, whereas the predator species diversity is seen not to significantly differ among the three grazing intensity treatments. Consequently, the relationship between arthropod species diversity and grazing intensity manifests a parabolic curve, which is consistent with the intermediate disturbance hypothesis in the context of grassland management, although these results may be pertinent to the management of other biologically similar ecosystems.

It is not surprising that plant species diversity is greater under moderate grazing compared to the other three treatments. Previous studies have reported that moderate grazing increases plant species diversity [53,54], supporting the intermediate disturbance hypothesis. The underlying mechanisms primarily underscore that moderate grazing decreases overall plant height and thus the intensity of light competition among plant species [28,55]. Moreover, moderate grazing permits the persistence of plant biomass sufficient to support plant diversity [56,57]. In this study, plant functional type diversity played an important role in regulating plant species diversity. In the nongrazing and light-grazing treatments, grass species (e.g., *Elymus nutans* and *Deschampsia caespitosa*) dominated the plant community, and forb species such as *Saussurea nigrescens* and *Potentilla discolor* dominated the heavy grazing treatment. In contrast, the moderate grazing treatment was dominated by both grass and forb species, allowing for the coexistence of a higher species diversity. Such a shift in dominant species might be due to the preference of yaks grazing to grass species [58].

In the study meadow, herbivore species diversity appears to be largely determined by plant species diversity, as indicated by the positive relationship between plant and herbivore species diversity (albeit statistically non-significant in 2021). One potential explanation for this observation is that the herbivorous insects in the study site are primarily leafhoppers, aphids, and thrips, such that many of these species are specialists feeding on specific plant taxa [59,60,61]. Moreover, the diversity of plant functional types (in the moderate grazing treatment) may facilitate the survival of generalist herbivore species requiring more than one functional type of plants [62]. For example, some grasshopper species are more generalized in feeding on both grass and forb species than some other grasshopper species [63]. In addition, grazing may directly facilitate herbivore species diversity. For example, it is reported that cattle grazing improves the abundance and diversity of grasshoppers species by leaving more plants preferred by grasshoppers [64].

Parasitoid species diversity appears to be partly determined by the herbivore species diversity in this study, as indicated by the positive correlation between herbivore and parasitoid species diversity. This correlation may be caused by the fact that most herbivorous insects (e.g., grasshoppers, sawfly larvae, and tephritid fly larvae) are potential hosts of parasitoids and because most parasitoids are host species specialists (e.g., tachinid flies and parasitoid wasps) [65,66,67]. Our findings are generally consistent (albeit statistically non-significant in 2021) with many previous studies [17,68]. Herbivores feeding on different plant functional types may be differentially preferred by parasitoids. In this study, the species that feed on forbs or both grass and forbs (e.g., moth larvae *Lacanobia contigua* and *Melanchra pisi*) seem to be more preferred by parasitoids than those feeding preferentially on grass species (e.g., moth larvae *Mythimna separata*). This observation is due to the caterpillars preferred by the parasitoids mostly prefer to feed on forbs [69]. However, in contrast to the expectation, the predator species diversity in this study did not peak in the moderate grazing treatment, perhaps because different functional types of predators occupy different niches in different grazing treatments. For example, web-building spiders tend to persist and occupy nongrazing and light-grazing treatments, where plants tend to be tall [70], whereas active-hunting spiders tend to persist in moderate and heavy grazing treatments, where caterpillars tend to be more abundant [71]. In addition, predatory beetles are generalists [72], preying on various herbivores in the different treatments used in this study.

It is worth noting that the positive relationships between plant and herbivore species diversity and between herbivore and parasitoid species diversity were not consistent between years. This could be attributed to the difference in climate conditions between years. Specifically, according to the meteorological records, the rainfall from July to August was 123 mm in 2021, ca. 30% lower than in 2020 (167 mm), resulting in a drought in the late growing season. This drought event might have differently impacted the species diversity of plants, herbivores, and parasites, thereby disrupting the relationships among them.

As predicted, saprophage species diversity decreased with increasing grazing intensity in this study. It is noteworthy that saprophages species richness and diversity primarily depend on the amount of both plant litter and soil humus [73]. Provided that both are often positively correlated [74], it can be inferred that saprophage species richness and diversity will increase with decreasing grazing intensity, which should increase with decreasing plant litter. By contrast, the species richness and diversity of coprophage species (i.e., dung beetles in this study) primarily depend on the amount of yak dung [75], which is expected to increase with increasing grazing intensity. Consistent with these expectations, the data indicate that coprophage species diversity decreases with increasing grazing intensity. Considering that the species diversity of both saprophage and coprophage species is at an intermediate level in the moderate grazing treatment, it is reasonable to speculate that moderate grazing increases detritivore species richness and diversity. However, the detritivore species diversity is inconsistent with this speculation, which can be attributed to the large variation in species evenness across the different treatments (data not shown). This inconsistency requires additional empirical examination, which in turn requires a much more robust experimental data set.

In summary, overall arthropod species diversity manifests a “hump” parabolic relationship with grazing intensity, consistent with the intermediate disturbance hypothesis. Importantly, the high abundance of parasitoids and predators may help prevent the outbreak of insect pests, stabilizing the biological community of our study pasture. This finding contributes to our current understanding that moderate grazing increases plant species diversity, soil organic matter accumulation, soil fauna diversity, soil microbe diversity, plant community stability, and soil microbial community stability, which collectively indicates that moderate grazing may be the most effective land use management practice to optimize the multi-functional services of pastures. Clearly, as in all site-specific studies, future studies should be conducted to explore the relevancy of the data and conclusions presented here.

## 5. Conclusions

The results presented here highlight the importance of the effects of grazing on arthropod functional groups. Moderate grazing intensity appears to be beneficial to overall arthropod diversity, which is consistent with the classical ecological theory that species diversity follows a unimodal response along the grazing intensity gradient and peaks at moderate grazing intensity. Importantly, the responses of different functional arthropod groups to grazing in this study are not consistent, indicating that the diversity of different functional groups should be considered when assessing the effects of grazing on biodiversity. Our results may have implications for pasture management. In the grasslands of west China, while overgrazing is not allowed to prevent local species loss, long-term fencing, which is currently used to restore degraded grasslands resulting from overgrazing, should be discouraged to maintain species diversity in biological communities.

## Figures and Tables

**Table 1:** Results of the one-way ANOVA of the effects of grazing on plant biomass, species richness, and diversity, and the effects of arthropod species groups. Significant effects of grazing intensity on indices (*p* < 0.05) are indicated in bold.

	2020		2021	
	*F*	*p*	*F*	*p*
Plant species richness	6.225	**0.017**	15.222	**0.001**
Plant species diversity	2.168	0.170	13.257	**0.002**
Plant biomass (g/m^2^)	93.480	**<0.001**	65.589	**<0.001**
Herbivore species richness	20.547	**<0.001**	8.379	**0.008**
Herbivore species diversity	7.921	**0.009**	4.287	**0.044**
Parasitoid species richness	9.003	**0.006**	5.959	**0.019**
Parasitoid species diversity	7.222	**0.012**	1.048	0.423
Predator species richness	1.101	0.404	1.555	0.274
Predator species diversity	1.338	0.329	2.094	0.179
Detritivore species richness	2.570	0.127	2.765	0.111
Detritivore species diversity	1.495	0.288	1.846	0.217
Coprophage species diversity	27.029	**<0.001**	9.666	**0.005**
Saprophage species diversity	4.121	**0.049**	1.095	0.405
Arthropod species richness	20.812	**<0.001**	18.825	**0.001**
Arthropod species diversity	11.168	**0.003**	4.797	**0.034**

**Table 2:** Effects of grazing intensity on the relative biomass of four plant functional types in 2020 and 2021. Data are provided as the means ± SE. The significant post hoc results (*p* < 0.05) based on the LSD test are shown with different lowercase letters. Significant effects of grazing intensity on indices (*p* < 0.05) are indicated in bold.

Year	Plant Functional Group	Grazing Intensity				*F*	*p*
		UG	LG	MG	HG		
2020	Grass	0.51 ± 0.09 ^b^	0.17 ± 0.04 ^a^	0.19 ± 0.05 ^a^	0.1 ± 0.01 ^a^	10.948	**0.003**
	Sedge	0.11 ± 0.04 ^a^	0.24 ± 0.03 ^b^	0.18 ± 0.05 ^ab^	0.18 ± 0.03 ^ab^	2.085	0.181
	Legume	0.01 ± 0.002 ^a^	0.03 ± 0.003 ^b^	0.03 ± 0.006 ^b^	0.04 ± 0.008 ^b^	6.503	**0.015**
	Forb	0.38 ± 0.06 ^a^	0.56 ± 0.07 ^b^	0.6 ± 0.07 ^b^	0.68 ± 0.04 ^b^	4.682	**0.036**
2021	Grass	0.4 ± 0.07 ^b^	0.25 ± 0.03 ^a^	0.25 ± 0.05 ^a^	0.14 ± 0.03 ^a^	5.123	**0.029**
	Sedge	0.3 ± 0.07 ^a^	0.24 ± 0.02 ^a^	0.21 ± 0.04 ^a^	0.23 ± 0.01 ^a^	0.974	0.451
	Legume	0.01 ± 0.004 ^a^	0.03 ± 0.002 ^b^	0.03 ± 0.007 ^b^	0.02 ± 0.005 ^ab^	2.438	0.139
	Forb	0.29 ± 0.07 ^a^	0.48 ± 0.04 ^b^	0.51 ± 0.09 ^b^	0.62 ± 0.03 ^b^	4.545	**0.039**

## Data Availability

Not applicable.
